# Annotations

**Published:** 1897-05-01

**Authors:** 


### Annotations.

### Mr. Ernest Hart and the Barrack Schools.

It is an old saying that the looker-on sees most of the game; and in the game now going on between the Guardians, the Departmental Committee, and the Local Government Board as to the workhouse children, Mr. Ernest Hart maintains that he, as a looker-on, occupying a peculiarly advantageous position, can see better and can get "a better grip of the situation" than those actually in the *mêlée*. A criticism of the report of the Departmental Committee has recently been published by order of a conference of managers, in which an endeavour is made to show that the whole movement was "organised, engineered, and inspired by Mr. Ernest Hart and his colleagues," and that "the chairman and some other members of the committee took their views blindly from him." There is no doubt that this pamphlet does show how largely the views entertained by Mr. Hart became impressed on the minds of the committee; but it is not to be forgotten that this body did not consist of babies, and if these views proved acceptable to those who had the witnesses before them, this would seem to speak something in favour of their truth. The fact is that in such a matter as this the onlooker's view is more likely than that of the school manager to be the true one. Take the simple question of ophthalmia. School managers, knowing well how quickly this disease gets the upper hand unless constant watchfulness is exercised, are well pleased at the great lessening of its prevalence which has taken place as the result of the care which has been expended upon it. The onlooker, however, takes quite a different view of the situation. To him it appears that a system under which all this care, this isolation, and this expenditure is necessary, to keep at bay such a malady as ophthalmia, is by that very fact condemned. The managers, no doubt, are proud that their endeavours have prevented the system of aggregation doing more harm than it has done. The onlooker, on the other hand, when he hears of people whose lives have been "largely spoilt by the disease," be they few or many, asks why such things should be. What is the remedy is another affair; but we can hardly doubt that in regard to the evils of the system the bystander has in this case seen most of the game, even though he has not visited the workhouses. Mr. Nettleship, at any rate, has seen the cases, and what he says is this: "The true conclusion to be drawn from the experience of the past is that so long as the present system continues, troublesome outbreaks of ophthalmia and other local contagious disorders, such as ringworm, will continue to occur from time to time, in spite of all such precautions as are practicable in schools of the Poor Law class."

### Plague.

That the plague is gradually dying out in Bombay seems evident, and, in fact, is only what must be expected. The probabilities are that the intensity of the epidemic is really diminishing in greater degree than is expressed by the official figures, for while on the one hand the efforts of the authorities are bringing a larger proportion of the cases to light, on the other the people are rapidly returning to the city, and thus the cases are spread among a larger population. At the same time it must not be forgotten that the returns have probably, all through the epidemic, had but the vaguest relation to actual facts. It seems as if the only possible way of obtaining a clue to the real condition of affairs is to consider only the total death-rate. However much doubt there may be as to the number of deaths from plague, there never need be any doubt as to the total of the dead from all causes. Judged from this standpoint it would appear that, while the epidemic is passing away from some quarters of the city, there are others in which it is still virulent. According to the figures given by the *Times of India* for April 9th the general death-rate for Bombay was 65 per 1,000, which is nearly twice the normal rate But there were districts in which the death-rate still stood as high as 103, 167, and even 236 per 1,000. The same considerations should be borne in mind in regard to the fatality of the disease. So far as the official figures are concerned, both as to Bombay and the other towns which have been affected, the proportion of deaths to cases has been enormous, but it is almost impossible to avoid the conclusion that these figures give an exaggerated view of the virulence of the disease. It is easier to hide cases than to hide deaths, and no doubt the apparent variations in the fatality in different towns and among different sects has been caused as much by variations in accuracy of registration as by variations in virulence. Even at the best, however, plague remains one of the most fatal of diseases.

### A Central Hospital Board for London.

A form of petition to His Royal Highness the Prince of Wales is being sent round for signature, praying for the formation of a Central Hospital Board "for the distribution of so important a public gift" as the Prince of Wales's Fund. That some committee will have to be formed to undertake the preparation of a scheme for the distribution of this money goes without saying. It is, however, unfortunate, to say the least, that this petition should adopt the phrase "Central Hospital Board," as indicating the body to undertake the work, and should state that "this proposal has already received the approval of a large number of the members of the medical profession and of many others interested in the welfare of the poor." The proposal which received the support here alluded to, and eventuated in the appointment of a general committee and an executive committee, the lists of which are enclosed along with the petition, was the formation of a Central Hospital Board for quite other purposes, and had nothing to do with the Prince of Wales's Fund. So far as concerns the proposal that a committee or board should be formed to distribute this important public gift we have nothing to urge except its complete lack of novelty and the underlying suggestion that all these things have not already occupied the mind of His Royal Highness. Our only feeling about this document is that we think it ought to be so worded that no mistake should arise as to its meaning, and that it should be impossible for anyone afterwards to say that those who had signed it had expressed themselves in favour of that form of Central Hospital Board which has been advocated by the authorities in Buckingham Street, whence this petition has its origin.

